# A retrospective study on the epidemiology and trends of road traffic accidents, fatalities and injuries in three Municipalities of Dar es Salaam Region, Tanzania between 2014-2018

**DOI:** 10.11604/pamj.2020.36.24.21754

**Published:** 2020-05-20

**Authors:** Francis Walugembe, Francis Levira, Balasubramanian Ganesh, Dickson Wilson Lwetoijera

**Affiliations:** 1 Nelson Mandela Institute of Science and Technology and Ifakara Health Institute, Arusha, Tanzania; 2 Environmental Health and Ecological Sciences Department, Ifakara Health Institute, Ifakara, Tanzania; 3 ICMR-National Institute of Epidemiology, R-127, Second Main Road, TNHB, Ayapakkam, Chennai-600 077, Tamil Nadu, India; 4 Environmental Health and Ecological Sciences Department, Ifakara Health Institute, Ifakara Tanzania

**Keywords:** Road traffic accidents, injury, fatalities, case fatality rates, Tanzania

## Abstract

**Introduction:**

Over 90% of injuries and deaths still occur in low and middle-income countries like Tanzania due to Road traffic accidents. Available literature indicates that Tanzania suffers massive human and economic losses every year from RTAs despite several interventions that have been made to curb this scourge. To gain an insight into the current state of RTAs we examined the pre- historical case fatality rates from RTAs in Ilala and two other municipalities (Kinondoni and Temeke) in Dar es Salaam Region, Tanzania.

**Methods:**

We conducted a retrospective study using the secondary data on road accidents from Road Accident Information System (RAIS) for the period 2014 to 2018.

**Results:**

A total of 6,772 road traffic injuries were reported between 2014 and 2018 and the study recorded the highest RTAs in the year 2014 as compared to the other years within the study period. The death rate from RTAs in Ilala Municipality alone was 36.4 per 100,000 population. About 28% of the total fatalities were recorded among the pedestrians, and there was a significant difference (P < 0.05) in the RTAs among the other road users.

**Conclusion:**

The study recommends the improvement of road transport infrastructure to ensure safety for all the road users by implementing the existing policies, strengthening the enforcement of existing legislation and introducing express penalties on a real-time basis. We encourage the use of this data to develop strategies in Tanzania that protect pedestrians and other vulnerable road users from RTAs.

## Introduction

Road traffic accidents (RTAs) are a major cause of global morbidity and mortality in developing counties [1], resulting in an estimated 20-50 million road traffic injuries (RTIs) and 1.35 million deaths per year [2]. The World Health Organization (WHO) in December 2018 has launched the global status report on road safety and it highlights that the road traffic injuries are now the leading killer of people aged between 5-29 years. Every day, almost 3,700 people are killed globally in road traffic crashes involving cars, buses, motorcycles, bicycles, trucks, or pedestrians. The burden is disproportionately borne by the pedestrians, cyclists and motorcyclists, in particular those living in the developing countries [2]. Overall, 93% of the world's fatalities on the roads occur in low-and middle-income countries, even though these countries have approximately 60% of the world’s vehicles than compared with the developed nations. Predictions are that deaths from non-communicable diseases such as RTAs will reach 49.7 million by the year 2020 [2]. Very recent reports show that in low and middle-income countries (LMICs), RTAs are responsible for economic losses of up to 65 billion dollars USD which is more than all development aid income combined [3]. Similarly, the risk of road deaths in these countries is estimated at 32.9 per 100,000 inhabitants as compared to just about 10.3 per 100,000in European countries [4, 5] or as compared with the Global road traffic death rate of 17.4 per 100,000 populations [6]. Likewise, in the year 2015, the UN General Assembly established Sustainable Development Goal 3.6 as the target of reducing road traffic deaths and injuries by 50% by 2020 [7]. Because, road traffic injuries are estimated to be the eighth leading cause of death globally for all age groups and the leading cause of death for children and young people. More people now die in road traffic crashes than from the global epidemic HIV/AIDS. Similar to the situation in most developing countries, roads are the dominant mode of transportation whereby 90-95% of the country’s goods and passengers are transported by roads. Unfortunately, this extensive use of roads accounts up to 16,211 road fatalities annually [8]. Moreover, the RTAs is associated with a significant economic loss of an estimated 800 USD and 3% of its Gross National Product (GNP) [3]. Of importance, several interventions such as driver training, public awareness campaigns, improvement of roads, increasing fines to RTA offenders, setting speed limits, deploying and regular inspection of vehicles by police have been implemented to address the challenge [5]. According to the global burden of diseases, injuries, and risk factors study (GBD), the road injuries are a unique cause of morbidity and mortality on the global landscape because unlike diseases and injuries for which there may be considerable lag between burden measurement, policy implementation and burden improvement, road injury burden can change rapidly if measures such as seatbelt laws, intoxicated driving laws and infrastructure improvements are implemented [9].

In November 2009 the first Ministerial Conference on Road Safety held in Moscow, Russian Federation, called on the United Nations to proclaim a Decade of Action for Road Safety [10]. In March 2010, the United Nations General Assembly adopted a resolution proclaiming 2011-2020 the Decade of Action for Road Safety and it was launched all over the world on the 11 May 2011. Road traffic injuries constitute a major health and development problem the world over but especially in the African Region. The landmark World Report on Road Traffic injury Prevention published in 2004 drew attention to the magnitude, causes and consequences of road traffic injuries. It also made recommendations on what needed to be done to stem the tide of the rising casualties from road traffic injuries. Since then many countries have taken measures to address the problem. The projections show that road traffic injuries will increase except decisive action is taken to tackle the problem [11]. Moreover, Road traffic accidents\crashes may be an everyday occurrence but they are both predictable and preventable, as illustrated by the large body of evidence on key risk factors and effective road safety measures that work in practice [12, 13]. However, despite the efforts of the government, concerned public and private sectors, the cost of road traffic accidents remains a big threat and burden to Tanzania’s economic capital and human resources. Dar es Salaam is one of the major cities and economic hubs in Tanzania with major road networks, and that necessitates a need for analysis of injuries and fatalities from RTAs in the region. The aim of the present research was to analyze the trends of fatalities/deaths and injuries from RTAs in three major municipalities of Dar es Salaam region-ilala, Temeke and Kinondoni during the years 2014 to 2018. We hypothesized that understanding these trends and patterns can help predict the need for additional services and resources and guide policy-makers on implementing appropriate and sustainable prevention strategies on a long-term basis.

## Methos

### Research methodology

#### Case definition

The operational case definition for a road traffic accident (RTA) or Road injury (RI)is interaction, as a pedestrian on the road, with an automobile, motorcycle, pedal cycle, or other vehicles resulting in bodily damage or death. According to the GBD, road injuries are an external cause of injury. Comparative analyses of secondary data on RTAs, and the number of registered vehicles in Tanzania from the period 2014 to 2018 in all three municipalities were conducted. A purposive sampling method adopted and the unit of analysis was all road accident reports Inilala, Kinondoni and Temeke municipalities of Dar es Salaam region, Tanzania for the period of 5 years from 2014 to 2018. Bartlett model [14] was adapted to establish sample size that was adequate for this study. The road traffic fatality rate was defined as the number of deaths at the scene of RTAs divided by the total number of people involved over the study period. The data was first explored through the use of graphical displays to help identify and analyze trends and patterns in the data and where two or more variables are looked at concurrently; comparative frequencies were used to establish the relationship between them. Descriptive analysis and correlation coefficients were also computed and interpreted. All statistical analyses were performed using BM SPSS Statistics Version 25. Ethical clearance was obtained from institutional Review Board ofifakara Health institute (IHI/IRB/No: 08-2019) and permission was also sought from theinspector General of Police Tanzania to access data from the Road Accident information System, a database of Tanzania Police Force, Department of Roads Traffic Division.

## Results

**Figure 1:**
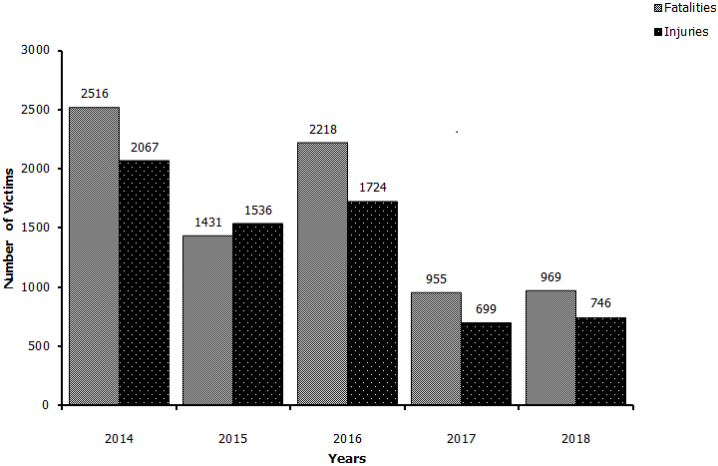
Road traffic injuries and fatalities in Ilala Municipality (2014-2018) as per Traffic Police Records, Dar es Salaam, Tanzania, 2018

**Figure 2:**
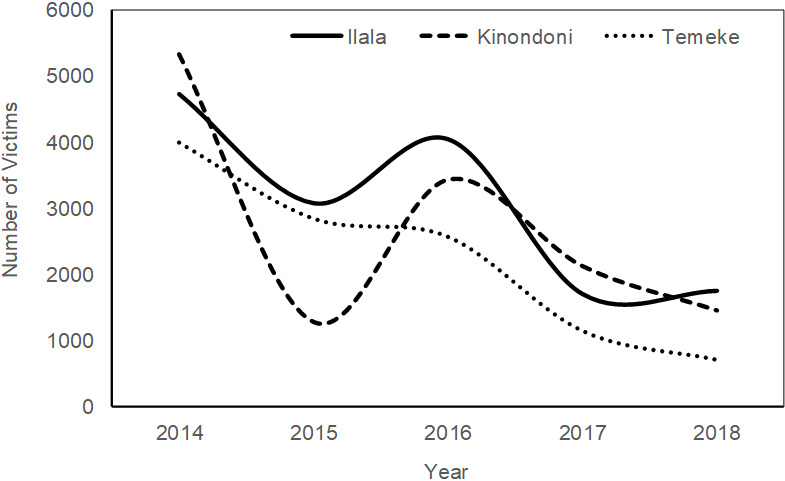
Variation of road accidents between Ilala, Kinondoni and Temeke municipalities in Dar es Salaam, Tanzania

**Figure 3:**
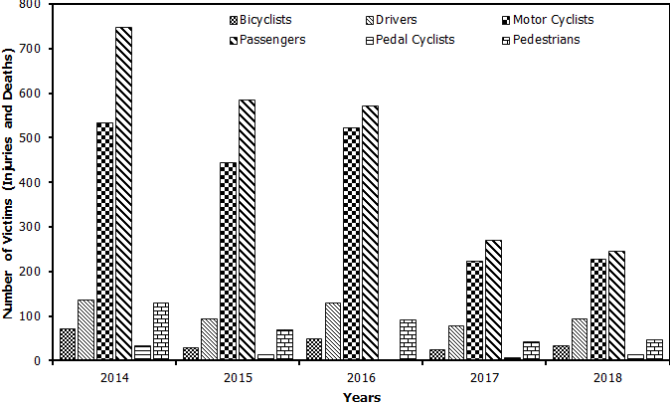
Total road accidents in Ilala Municipality from 2014 to 2018

**Figure 4:**
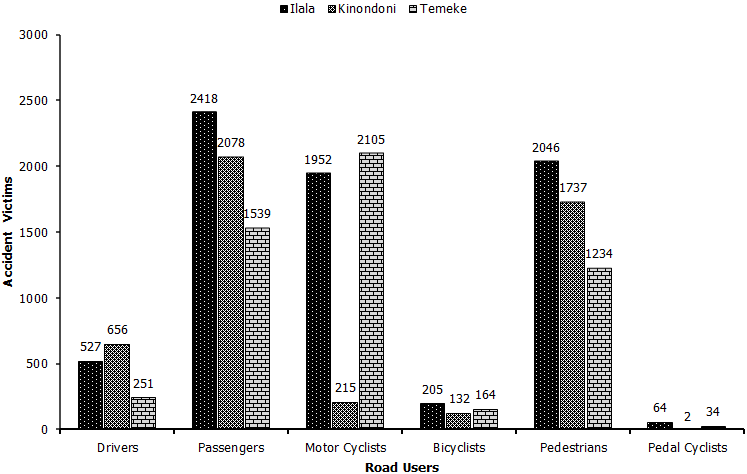
Total road accidents for the road users in Ilala, Kinondoni and Temeke municipalities in Dar es salaam, Tanzania (2014-2018); Source: Traffic Police Records, Dar es Salaam, Tanzania (2018)

The data on road traffic injuries and deaths in Ilala municipality between the year 2014 and 2018 is reflected in Figure 1. A total of 6,772 road traffic injuries were reported at Ilala municipality in Tanzania between 2014 and 2018. The year 2014 recorded the highest number of road fatalities of 2,516 compared to the year 2017 which recorded the last number of road injuries (699) within the municipality. A close comparative analyses of data retrieved from the Road Accident information System indicates a significant difference (P<0.05) in the number of road accident among the municipalities with Ilala Municipality registering the highest number of accidents (15,301) compared to 13,613 and 11,292 in Kinondoni and Temeke respectively. Nevertheless, there was a marked decline in the number of RTAs in all the three municipalities as shown in Figure 2. This study also sought to determine the groups of road users most affected by RTAs. According to findings from the Tanzania Police Force Headquarters on Ilala, the total number of road accidents from the year 2014 to 2018 was 2,046 for both passengers and pedestrians, 1,952 for motorcyclists; 527 for drivers; 205 for bicyclists; and only 64 for pedal cyclists (Figure 3). Comparative evaluation of the most affected groups of road users ini Iala, Kinondoni and Temeke revealed differing trends (Figure 4). The results showed that passengers and pedestrians, as well as drivers were the most affected road users in all three municipalities. Motorcyclists were mostly affected in Temeke compared to Kinondoni andilala municipalities. The bicyclists and pedal cyclists were generally the least affected of all the other types of road users. However, the obtained data still showed that pedal cyclists were more affected in Ilala than the other two municipalities. Table 1 provides the case-fatality rates for the different road users affected by road accidents in the three municipalities in Dar es Salaam as from 2014 to 2018. It was evident that passengers had the highest case-fatality rate (15.93%) in Ilala, while Pedestrians had the highest case fatality ratesin Temeke (10.45%) as well asin Kinondoni (16.93%).

**Table 1:** Case-fatality rates for the road users affected by road accidentsin Dar es Salaam Municipalities (2014-2018)

Municipality	Drivers	Passengers	Motorcyclists	Bicyclists	Pedestrians	Pedal Cyclists
Ilala	4.74%	15.93%	6.45%	7.80%	9.19%	7.81%
Temeke	5.98%	4.81%	5.70%	10.36%	10.45%	5.88%
Kinondoni	10.21%	8.33%	14.57%	13.63%	16.93%	0.00%

### Analysis of relationship between road traffic accidents and the number of registered motor vehicles in Tanzania

Table 2 presents summary of model statistics which was used to assess the exact nature of the relationship between the road traffic accidents and the number of registered motor vehicle by the use of correlation coefficients. Specifically, the R which represents the correlation coefficient of value 0.944 shows a strong positive relationship between road traffic accidents and the number of vehicles registered. The coefficient of determination (R-square) which is a measure of the explanatory power of the model indicate that the number of registered vehicles is able to account for or explain 89.1% of the changes in the number of road traffic accidents in Tanzania. This also implies that there are other variables or factors that have effect on road traffic accidents which of course are not included in this model in the neighborhood of about 20.9%. Table 3 reports the regression coefficients and other vital statistics such as standard errors of the coefficients, the t values and the p values. The regression model, which establishes the relationship between total annual accidents and number of registered vehicles is thus given as Y = 647000 + 0.019X1, where Y is the total number of accidents in a year and X1 being the total number of registered vehicles in a year. From the regression model obtained, the value of 647000 is interpreted to be the total number of yearly accidents when the total number of registered vehicles is set to zero and all other factors are held constant, whilst the coefficient of X1 of 0.019 is the rate or magnitude of change in the number of accidents as a result of a unit change in the number of registered vehicles. Its positive sign is an indication of the fact that there is a positive association between road traffic accidents and the number of registered vehicles.

**Table 2:** Summary of Model statistics

Statistic	Value
R	0.944
R-Square	0.891(89.1%)
R-Square (Adjusted)	0.746(74.6%)
Standard Error	3734.635

Predictors: Constant, Number of vehicles registered; Dependent variable: Road Traffic Accidents

**Table 3:** Summary of regression coefficients municipalities (2014-2018)

Predictor	Coeff.	SE Coeff.	T-value	P-value
Constant	647000	3734	15.2632	
Vehicle(X1)	0.019	0.0038	6.9935	0.016

Predictors: Constants, number of vehicles registered; Dependent variable: Road Traffic Accidents

## Discussion

The aim of this research was not only to enhance the understanding and awareness of RTAs but also to inform decision-making organs and policy-makers of the consequences of the neglected public health problem of road carnage. The number of accidents drastically reduced from 4,045 in 2016 to 1,701 in 2018 probably because of increased enforcement of traffic regulations and the presence of traffic police officers on the roads as well as intensified road safety campaigns by the national road safety council [4]. For instance, the first and the second road safety campaigns launched in August 2016 to February 2017 and July to December were lauded for this reduction in accidents [15]. The collaboration of the police force with other bodies such as Surface and Marine Transport and regulatory authority [4] could also have contributed to the reduction by ensuring that all road users abided by the traffic regulations. Reports by Mujalli (2018) [16], agree that most measures taken during the period 2010 to 2013 proved to be successful in reducing the RTAs. When interpreting Tanzanian Traffic Police data, it should be noted that Tanzania, like many other developing countries, does not have a real-time unified national level reliable system for recording injuries or reporting systems at a later stage. For instance, the World Health Organization, in its Global Status Report for road safety [17], classifies Tanzania as a country without eligible death registration data. Research shows that for every road traffic fatality, at least 20 people sustain non‐fatal injuries [12]. Thus, the numbers in the traffic police data would be far much higher. The results from this study agree with that of the other published reports that showed there is a global reduction in death rates from RTAs in Africa region or worldwide [17, 18]. Further reports indicate that there are countries which have indeed successfully managed to reduce the number of deaths on roads, although this may not be so in all cases [19, 20]. Nevertheless, although the present study showed that RTAs have significantly reduced in the study areas in Tanzania, a very recent report by Kazeem (2019) [21] reveals that death rates from RTAs in Sub-Saharan Africa are still higher than anywhere else in the world. The study investigated the groups of road users mostly affected by RTAs in Ilala as well as Kinondoni and Temeke municipalities in Dar es Salaam. Some indication of the order of priorities in road accident prevention could be gained from the distribution of accident injuries among the various road user classes. Furthermore, the findings of this study showed that the case-fatality rate for passengers was high in Ilala compared to Kinondoni and Temeke municipalities. Although, the case-fatality rates for drivers, pedestrians, bicyclists and motorcyclists were higher in Kinondoni compared to Ilala and Temeke municipalities where fatality rates were high mostly for passengers. The results showed that 33.57% (2,418) of the total injuries from RTAs occurred to the passengers. This could be attributed to the fact that passengers constitute the majority of vehicle users [22], and public transport is the daily routine for most of poor Africans and older reports agree that high percentage of passenger fatalities are associated with the utilization of public transport [23]. Of greater concern was the large number of pedestrians who constituted the second-highest numbers (28.41%) of the injured and the dead from RTAs. Although disturbing, this finding has consistently been reported in many developing countries (World Health Organization, 2015). Previous studies have established that most of the roads in Dar es Salaam and other parts of Tanzania do not have side pavements for pedestrians or cyclists and sometimes all road users have to crowd on the road [22, 24] which could be the fueling factor for injuries to pedestrians from RTAs. Additionally, there are few pedestrian crossing areas, which should be of particular concern to schools in close proximity to highways [22, 25]. Public awareness on road use is fairly low and pedestrians are less likely to use walking pavements even when they are available [22]. Research from Brazil supports this hypothesis that the lack of pedestrian lanes is associated with high-risk features [26]. Likewise, 27.10% (1952) of injuries occurred to motorcyclists. Some reasons which have been put forward concerning this in the Dar es Salaam region in a very recent study include over speeding, reckless driving, traffic violations and driving under the influence of alcohol [27]. Dar es Salaam being a hugely populated region with more than 5 million people has over 300,000 registered motorcycles which are preferred as taxis especially where conventional transportation is uneconomical or physically impossible due to poor road infrastructure [28-30]. Studies show that motorcycle injuries are among the leading causes of deaths and the main victims are usually motorists, passengers, and pedestrians [13]. This author reiterates that the risk of dying from a motorcycle accident is 20 times higher than from a motor vehicle. Road users who were least affected by RTAs in all three municipalities were the drivers, bicyclists, and pedal cyclists. In a similar study carried out by Museru *et al.* (2002) [22], similar findings were reported where just about 7% and 3% of RTAs were attributed to pedestrians and pedal cyclists respectively.

The present study investigated the relationship between RTAs and the number of registered vehicles. Moreover, this study showed a strong and positive correlation between the number of registered vehicles and the number of RTAs within all three municipalities. This could probably mean that RTAs are strongly related to the large number of registered vehicles such as buses, pickups, and Lorries [10]. These vehicles are often overloaded and are often operated under pressure to achieve their daily targets which contributes to the high casualty rates as the buses are often involved in reckless driving while competing for passengers with that of their parallel service providers [5, 7]. The coefficient of determination (R2) which is a measure of the explanatory power of the model indicated that the number of registered vehicles is able to account for or explain 89.1% of the changes in the number of road traffic accidents in the study areas. This implies that there are other variables or factors that contribute to about 10.9% of RTAs which are not included in this model.

## Conclusion

To our knowledge, this is one of the first studies to assess regional level RTAs, fatalities, injuries, incidence and mortality rates in three municipalities of Dar es Salaam Region, Tanzania over a five-year period. This study points out the factors leading to injury severity of RTAs and most at-risk road users in urban Dar es Salaam. In general, prevention strategies in Tanzania have been mainly implemented for vehicle occupant, whereas little attention has been paid to other road users. However, injuries to pedestrians, bicyclists, motor cyclists and pedal cyclists remain a major public health concern. Laws and regulatory frameworks should be formulated and enforced promptly to avoid losses caused by the occurrence of an accident. RTAs require a collaborative approach from different sectors so as to address RTAs in a holistic manner. Pedestrian’s walkways and pedal-cyclists lanes should be factored in road design. The government, the police, the health personnel and general public should be incorporated into preventive measures to be formulated. Road safety professionals should be trained, to monitor the magnitude, severity and burden resulting from RTAs in Dar es Salaam to counteract the paucity of evidence occasioned by insufficient data handling skills. According to the GBD on Morbidity and mortality from road injuries, showed clearly that despite improvements in mortality, road injuries remain critically important cause of morbidity and mortality globally, and more research is needed to better measure and understand how road injuries can be prevented, particularly in developing economies. Investing in preventative measures as well as ensuring that victims of road injuries have access to first response trauma and medical care could help drive improvements in road injury burden in the future [9]. To reduce RTAs and its associated fatalities, the government should improve road infrastructure to facilitate easy movement of all road users, for example by having side-walks to ease movement of pedestrians, and enforce regulations on consistent use of seat belts, avoid over-speeding and overloading of passengers especially in public buses, and conducting regular road safety education campaigns. This study was limited to the three original municipalities of Dar es Salaam and data available in the Road Accident information System at the Regional Traffic Department and could have inconsistent reporting. City planning should therefore incorporate all road users in mind and should focus more on the behavior and the setting. Law enforcement officers should also be trained on different important aspects of road safety. This study also elucidates the RTAs burden and establishes a baseline, which helps to inform future work, with the overall goal of reducing RTAs incidence and mortality in Tanzania.

### What is known about this topic

- Road traffic accidents are a major public health concern in Tanzania killing 32.9 per 100,000 people per annum;
- Community engagement and sensitization of all road users is vital for reduction of RTAs;
- Although several interventions have been implemented to reduce road carnage in Tanzania, fatalities are still high and above the recommended 20 per 100,000 people by World Health Organization.

### What this study adds

- This study generates empirical evidence that highlights the key challenges and possible mitigations in reducing RTAs in urban Dar es Salaam;
- Availability of empirical data reveals the magnitude of the problem thus helping to identify the risk factors and target groups so that a scientific approach to prevention and control of RTAsican be prepared;
- The study findings have a potential to guide planning and implementation of road safety campaigns by National Road Safety Council in Tanzania.

## Competing interests

The authors declare no competing of interests.
